# Glibenclamide in Aneurysmal Subarachnoid Hemorrhage: A Systematic Review and Meta-Analysis of Randomized Controlled Trials

**DOI:** 10.7759/cureus.90067

**Published:** 2025-08-14

**Authors:** Amal A K Alsubaiei, Abdullah M Alharran, Abdulrahman K Alfailakawi, Ahmad A Alahmad, Malek K Hasan, Eid Y AlOthainah, Plamen Penchev

**Affiliations:** 1 Department of Medicine and Surgery, Arabian Gulf University, Manama, BHR; 2 College of Medicine and Medical Sciences, Arabian Gulf University, Manama, BHR; 3 Department of Medicine, Royal College of Surgeons in Ireland, Dublin, IRL; 4 Department of Medicine and Surgery, Royal College of Surgeons in Ireland, Dublin, IRL; 5 Faculty of Medicine, Kuwait Institute for Medical Specializations, Kuwait City, KWT; 6 Faculty of Medicine, Medical University of Plovdiv, Plovdiv, BGR

**Keywords:** aneurysmal subarachnoid hemorrhage, bleeding, death, glibenclamide, systematic review and meta-analysis

## Abstract

Aneurysmal subarachnoid hemorrhage (aSAH) is a critical neurological condition with high morbidity and mortality. This study aims to evaluate the effectiveness of glibenclamide through a systematic review and meta-analysis of randomized controlled trials (RCTs). We performed a systematic review and meta-analysis according to Preferred Reporting Items for Systematic Reviews and Meta-Analyses (PRISMA) guidelines, focusing on RCTs assessing glibenclamide's safety and efficacy in aSAH patients. A comprehensive search of multiple databases was conducted, with studies published until January 2025. Statistical analysis was conducted using RevMan software (The Cochrane Collaboration, London, UK) to determine the effect of glibenclamide on clinical outcomes. A total of four RCTs with 290 patients were included. No significant differences were observed between the glibenclamide and control groups in delayed cerebral ischemia (DCI) (risk ratio (RR): 0.58, 95% confidence interval (CI): 0.31 to 1.10, P = 0.09, I² = 0%), hydrocephalus (RR: 1.65, 95% CI: 0.97 to 2.71, P = 0.06, I² = 0%), death (RR: 0.88, 95% CI: 0.50 to 1.56, P = 0.66, I² = 0%), or hypoglycemia (RR: 3.53, 95% CI: 1.00 to 12.54, P = 0.05, I² = 0%). Additionally, no significant differences were found in modified Rankin Scale (mRS) scores at six months (mean difference (MD): -0.45, 95% CI: -1.11 to 0.20, P = 0.18, I² = 0%) or at three months (MD: 0.06, 95% CI: -0.60 to 0.72, P = 0.86, I² = 0%). This meta-analysis found no significant advantage of glibenclamide over the control group in improving outcomes for aSAH patients. Despite the generally low risk of bias in the studies, potential publication bias should be considered when interpreting these findings.

## Introduction and background

Aneurysmal subarachnoid hemorrhage (aSAH) represents a severe neurological emergency characterized by the rupture of an intracranial aneurysm, resulting in the extravasation of blood into the subarachnoid space. This condition is associated with considerable morbidity and mortality, with case fatality rates reported between 25% and 50% [1]. The pathophysiological mechanisms underlying aSAH are multifaceted, encompassing the immediate effects of subarachnoid blood, which can precipitate delayed cerebral ischemia (DCI) and cerebral vasospasm. These sequelae are among the principal contributors to unfavorable clinical outcomes [2,3]. Despite advancements in both surgical and medical management, patient prognoses remain challenging, highlighting the imperative need for innovative therapeutic strategies.

Glibenclamide, a sulfonylurea class drug primarily employed in diabetes management, has emerged as a promising neuroprotective agent in the context of aSAH. Its therapeutic action is attributed to the inhibition of the Sur1-Trpm4 channel, a critical mediator in the pathophysiological cascade following subarachnoid hemorrhage [4]. Evidence from preclinical studies indicates that glibenclamide may attenuate the deleterious effects of subarachnoid blood, thereby reducing the incidence of delayed cerebral ischemia and enhancing clinical outcomes [4]. Given the substantial burden associated with aSAH, conducting randomized controlled trials (RCTs) to evaluate the efficacy and safety of glibenclamide is both timely and essential.

Recent systematic reviews and meta-analyses underscore the necessity of exploring pharmacological interventions in aSAH, including glibenclamide. These studies emphasize the importance of robust clinical trials to establish evidence-based treatment guidelines aimed at improving patient outcomes. The present systematic review and meta-analysis seek to synthesize the existing body of literature on glibenclamide in the setting of aSAH, with a specific focus on RCTs. By consolidating data across multiple studies, this review aims to elucidate the therapeutic potential of glibenclamide, particularly in terms of its effects on clinical outcomes. The findings are expected to contribute to the ongoing development of optimized treatment strategies for this life-threatening condition.

## Review

Methodology

Material and Methods

We performed a systematic review and meta-analysis following the guidelines outlined in the Preferred Reporting Items for Systematic Reviews and Meta-Analyses (PRISMA). The study protocol has been registered on PROSPERO (ID: CRD42025638375).

Search Strategy

We conducted a systematic search across MEDLINE (PubMed), Web of Science, Central, Google Scholar, and Scopus, using comprehensive and inclusive terms, including Medical Subject Headings (MeSH) terms, for keywords such as "glibenclamide" and "aneurysmal subarachnoid hemorrhage," up to January 2025. There were no restrictions on language or country. Detailed search strategies are provided in the Appendices.

Search results were managed using EndNote 21® (Clarivate, London, UK), where duplicates were removed, and the remaining studies were transferred to an Excel sheet (Microsoft Corp., Redmond, WA) for screening. Two authors independently reviewed the titles and abstracts of the studies based on the eligibility criteria, with discrepancies resolved by a third author. Full-text articles of the eligible studies were then independently screened by the same two authors, and any disagreements during this stage were resolved through discussion. Additionally, we performed a manual search by reviewing the reference lists of the included studies and any relevant published reviews.

Eligibility Criteria

The inclusion criteria for this study were as follows: (A) studies were limited to those conducted on human subjects, (B) the primary focus had to be on evaluating the efficacy or safety of glibenclamide, (C) only articles published in peer-reviewed journals were considered, and (D) studies needed to provide sufficient data for both qualitative and quantitative analysis. The exclusion criteria were as follows: (A) studies evaluating interventions other than glibenclamide were excluded, and (B) reviews, case reports, editorials, books, and comments were not included in the analysis.

Study Selection and Data Extraction

Data extraction was carried out by two authors using a standardized Excel data extraction form. The final decision regarding the inclusion of each study was reached through consensus among all authors. Key data extracted from the included studies included the first author, study design, country, total participants, dose, follow-up period, inclusion criteria, body mass index (BMI), hypertension, age, gender, and Hunt-Hess grade.

Quality and Evidence Assessment

We utilized the Cochrane Risk of Bias (RoB) assessment tool, as described in the Cochrane Handbook for Systematic Reviews of Interventions version 6.0. This tool evaluates five domains of bias: performance bias, selection bias, detection bias, reporting bias, and attrition bias. Each randomized controlled trial (RCT) was classified as having a high, unclear, or low risk of bias based on these domains. The quality of evidence was evaluated using the GRADE system (Grading of Recommendations, Assessment, Development, and Evaluations). Randomized trials were initially classified as high quality but could be downgraded due to factors such as bias, inconsistency, indirectness, imprecision, or other concerns. Conversely, evidence could be upgraded if there was a substantial effect size or if confounding factors likely diminished the observed impact. Each outcome was ultimately rated as high, moderate, low, or very low quality.

Statistical Analysis

We used RevMan software version 5.4.1 (The Cochrane Collaboration, London, UK) for the analysis. Data such as events and total of adverse events, and mean and standard deviations (SD) of modified Rankin Scale (mRS) score were extracted. The results were presented in mean or risk ratios (RRs), along with the confidence intervals (CIs). Heterogeneity was assessed using the inconsistency index (I²) and the Chi-squared (X²) test. The I² statistic was used to quantify the degree of variation in study outcomes, with values exceeding 50% indicating substantial heterogeneity and values above 90% signifying major heterogeneity. Statistical significance was defined as a P-value of less than 0.05.

Results

Search Results

**Figure 1:**
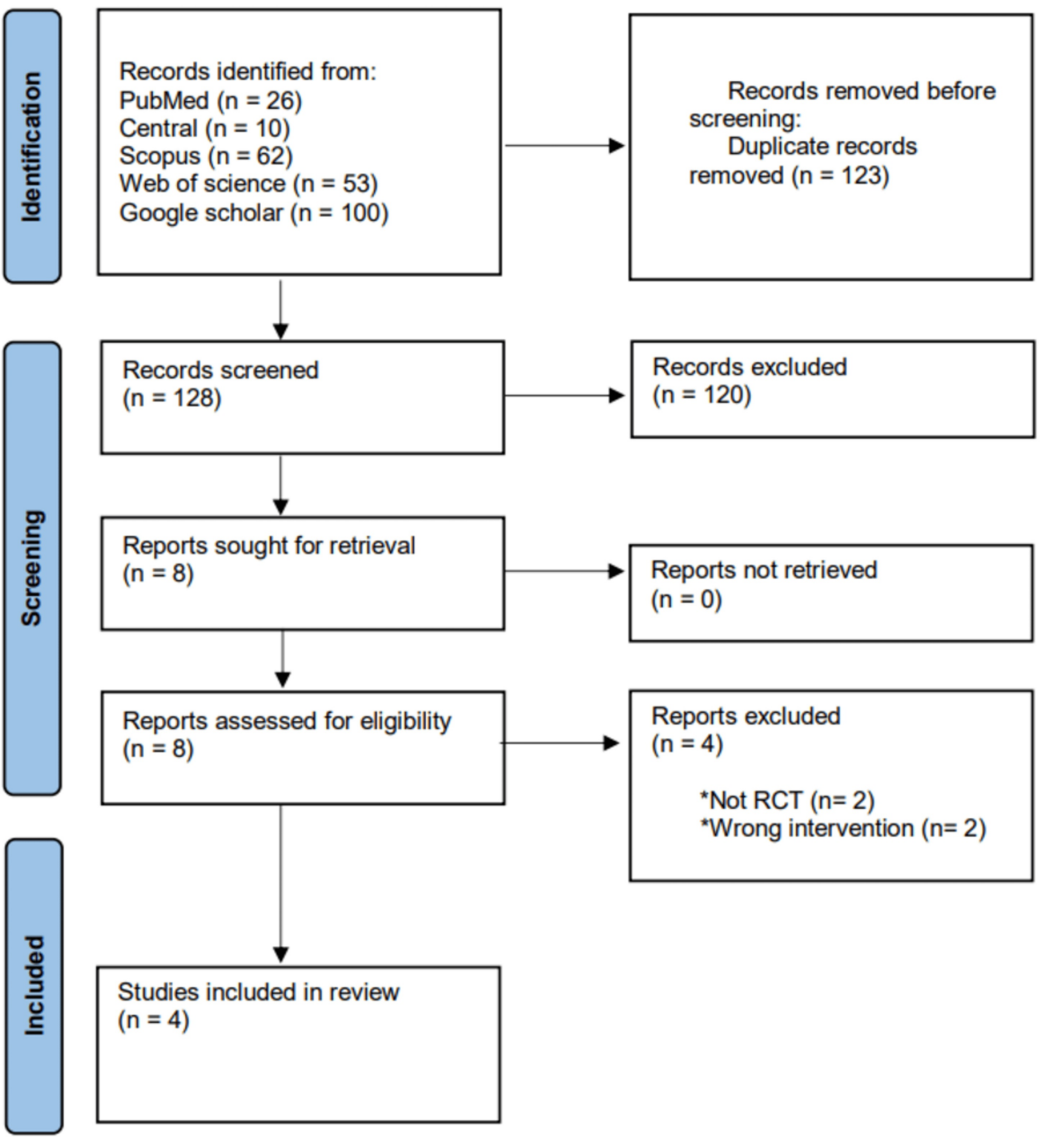
PRISMA flowchart of the selection process PRISMA: Preferred Reporting Items for Systematic Reviews and Meta-Analyses, RCT: randomized controlled trial

A total of 251 articles were extracted from PubMed, Central, Scopus, Web of Science, and Google Scholar. After the removal of duplicates, 128 papers have been initially screened (title/abstract). Subsequently, eight papers have been screened (full text), of which only four randomized controlled trials have met the inclusion criteria and were included in both the systematic review and meta-analysis, as shown in the PRISMA flow diagram [1,5-7]. The remaining four studies were excluded because two were not randomized controlled trials and two investigated the wrong intervention (Figure 1).

Characteristics of the Included Studies

The included studies collectively examined 290 patients with aneurysmal subarachnoid hemorrhage (aSAH) across four randomized controlled trials (RCTs) conducted in Brazil and China. Patient populations varied in size, with sample sizes ranging from 45 to 111 participants per study. The intervention arms involved oral administration of glibenclamide at different doses (ranging from 3.75 mg to 15 mg daily), with durations of treatment spanning 7-21 days. Follow-up periods varied, including 10 days, 90 days, and six months. Participants were generally adults aged 18-74 years who were diagnosed with radiologically confirmed aSAH within 48-96 hours of ictus (Table 1).

**Table 1:** Characteristics of the included studies RCT: randomized controlled trial, aSAH: aneurysmal subarachnoid hemorrhage, mRS: modified Rankin Scale, NSE: neuron-specific enolase, S100B: soluble protein 100B, QoL: quality of life

Study	Study design	Country	Total participants	Trial arm		Glibenclamide dose	Follow-up	Main inclusion criteria	Outcome measures	Conclusion
				Intervention	Control					
da Costa et al. (2022) [7]	RCT	Brazil	78	Glibenclamide	Placebo	5 mg orally daily for 21 days	6 months	Radiologically confirmed aSAH, aged 18-70 years, presented within 96 hours of ictus	Mortality and functional status at discharge and 6 months, evaluated using the mRS	Glibenclamide was not associated with better functional outcomes after aSAH, and mortality and delayed cerebral ischemia rates were similar compared with placebo.
Feng et al. (2024) [1]	RCT	China	56	Glibenclamide	Placebo	15 mg orally daily for 10 days	10 days	aSAH, age 18 or older, surgery within 72 hours, Hunt-Hess grade 2 or higher	Proportion of patients achieving the Subarachnoid Hemorrhage Early Brain Oedema Score dichotomy (defined as 0-2) at the 10-day post-medication	Oral administration of high-dose glibenclamide significantly reduced radiological assessment of cerebral edema after 10 days of medication, but it was associated with a higher incidence of hypoglycemia.
Lin et al. (2024) [6]	RCT	China	111	Glibenclamide	Standard care	3.75 mg orally daily for 7 days	90 days	aSAH within 48 hours of onset, age 18-74 years	Levels of serum NSE and S100B	Treating patients with early aSAH with oral glibenclamide did not decrease levels of serum NSE and S100B and did not improve the poor 90-day neurological outcome.
Windlin et al. (2025) [5]	RCT	Brazil	45	Glibenclamide	Placebo	5 mg orally daily for 21 days	6 months	aSAH, age between 18 and 70 years, definitive treatment within 96 hours of ictus	Cognitive performance, QoL, and emotional aspects	Glibenclamide did not improve cognitive performance, QoL, and emotional aspects after 6 months of follow-up of aSAH survivors.

The average age of participants ranged from 49.9 to 61.8 years, with gender distribution showing a higher proportion of female participants in most groups. Body mass index (BMI) was reported in two studies, with mean values ranging from 24.2 to 25.6. The Hunt-Hess grade, where available, ranged from 2 to 4, with a median of 3 across two studies, reflecting moderate to severe initial neurological status. Hypertension prevalence was reported in two studies, ranging from 50% to 82.1% (Table 2).

**Table 2:** Characteristics of the included patients SD: standard deviation, BMI: body mass index

Study	Group	Number of participants in each group	Age (years), (mean ± SD)	Gender (male/female)	BMI (mean ± SD)	Hunt-Hess grade	Hypertension (number (%))
da Costa et al. (2022) [7]	Glibenclamide 5 mg/day	n = 38	53.6 ± 11.6	6/32	Not reported	3 (2-4)	Not reported
	Placebo	n = 40	52.7 ± 11.3	13/27	Not reported	3 (2-4)	Not reported
Feng et al. (2024) [1]	Glibenclamide 15 mg/day	n = 28	61.8 ± 11.6	12/16	24.3 ± 3.6	3 (3-4)	23 (82.1)
	Placebo	n = 28	59.1 ± 12.6	17/11	25.6 ± 4.7	3 (3-4)	18 (64.3)
Lin et al. (2024) [6]	Glibenclamide 3.75 mg/day	n = 57	56 (50, 65)	28/29	24.5 (22.8, 26.1)	Not reported	35 (61)
	Standard care	n = 54	55 (48.25, 62.75)	25/29	24.2 (22.35, 26.32)	Not reported	27 (50)
Windlin et al. (2025) [5]	Glibenclamide 5 mg/day	n = 23	49.9 ± 11.9	2/21	Not reported	Not reported	Not reported
	Placebo	n = 22	50.0 ± 12.1	10/12	Not reported	Not reported	Not reported

Quality and Evidence Assessment

**Figure 2:**
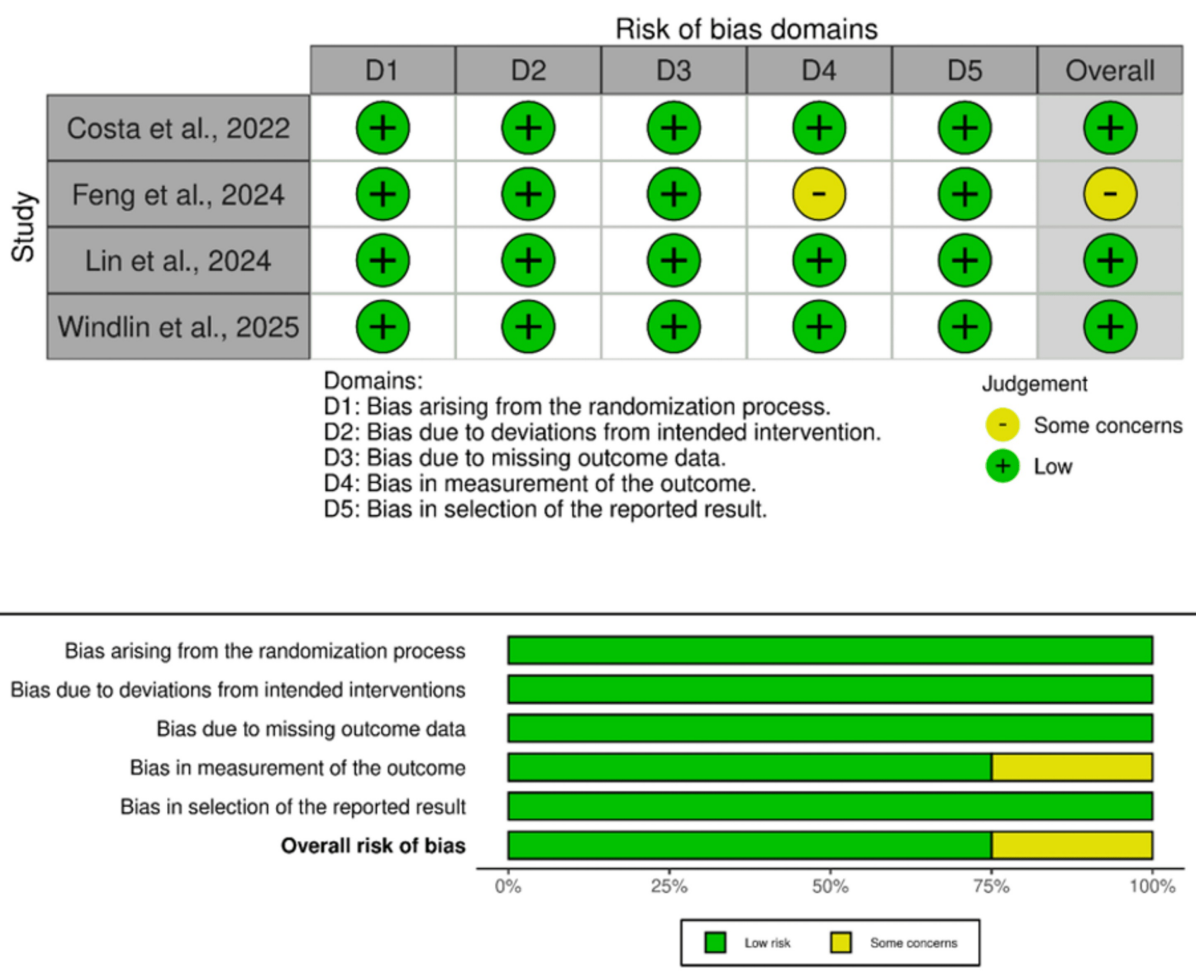
Quality assessment of the randomized controlled studies using the RoB 2 tool [1,5-7] RoB: Risk of Bias

We assessed the quality of the included studies using the RoB 2 tool. Out of four studies, three were considered to have low risk of bias, indicating high quality. One study was considered to have a moderate risk of bias, indicating moderate quality due to some concerns in reporting the outcomes (Figure 2). According to the GRADE assessment, the quality of most outcomes were moderate (Appendices).

Meta-Analysis

**Figure 3:**
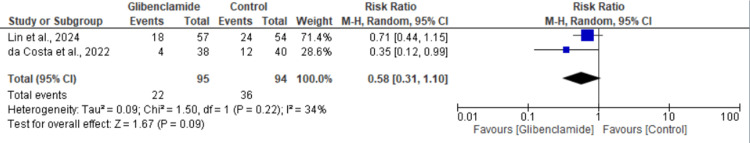
Meta-analysis of the risk of delayed cerebral ischemia between control patients and patients on glibenclamide [6,7]

Delayed cerebral ischemia: Two studies assessed the risk of delayed cerebral ischemia. Our meta-analysis demonstrated no significant difference between the glibenclamide and control group in delayed cerebral ischemia (RR: 0.58, 95% CI: 0.31 to 1.10, P = 0.09). The heterogeneity among studies was zero for delayed cerebral ischemia (I² = 0%) (Figure 3). 

**Figure 4:**
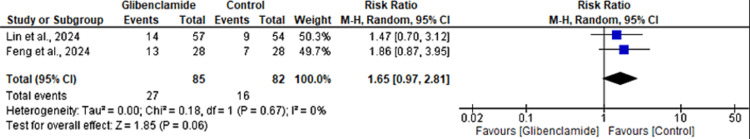
Meta-analysis of the risk of hydrocephalus between control patients and patients on glibenclamide [1,6]

Hydrocephalus: Two studies evaluated the risk of hydrocephalus. Our meta-analysis showed no statistically significant difference between the glibenclamide and control groups in the incidence of hydrocephalus (RR: 1.65, 95% CI: 0.97 to 2.81, P = 0.06). Heterogeneity across studies was low (I² = 0%) (Figure 4).

**Figure 5:**
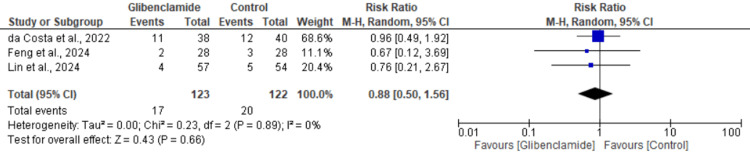
Meta-analysis of the risk of death between control patients and patients on glibenclamide [1,6,7]

Death: Three studies assessed the risk of death. Our meta-analysis demonstrated no significant difference between the glibenclamide and control group in death (RR: 0.88, 95% CI: 0.50 to 1.56, P = 0.66). The heterogeneity among studies was zero for death (I² = 0%) (Figure 5).

**Figure 6:**
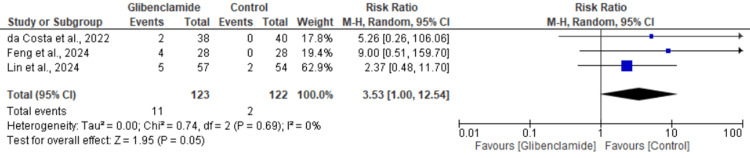
Meta-analysis of the risk of hypoglycemia between control patients and patients on glibenclamide [1,6,7]

Hypoglycemia: Three studies assessed the risk of hypoglycemia. Our meta-analysis demonstrated no significant difference between the glibenclamide and control group in hypoglycemia (RR: 3.53, 95% CI: 1.00 to 12.54, P = 0.05). The heterogeneity among studies was zero for hypoglycemia (I² = 0%) (Figure 6).

**Figure 7:**
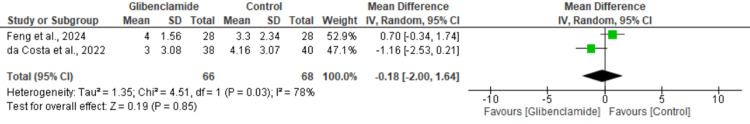
Meta-analysis of the mRS at discharge between control patients and patients on glibenclamide [1,7] mRS: modified Rankin Scale

mRS at discharge: Two studies assessed mRS at discharge. Our meta-analysis demonstrated no significant difference between the glibenclamide and control group in mRS at discharge (MD: -0.18, 95% CI: -2.00 to 1.64, P = 0.85). The heterogeneity among studies was high for mRS at discharge (I² = 78%) (Figure 7).

**Figure 8:**
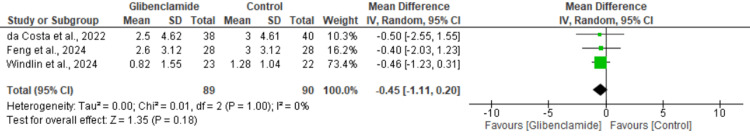
Meta-analysis of the mRS at six months between control patients and patients on glibenclamide [1,5,7] mRS: modified Rankin Scale

mRS at six months: Three studies assessed mRS at six months. Our meta-analysis demonstrated no significant difference between the glibenclamide and control group in mRS at six months (MD: -0.45, 95% CI: -1.11 to 0.20, P = 0.18). The heterogeneity among studies was zero for mRS at six months (I² = 0%) (Figure 8).

**Figure 9:**
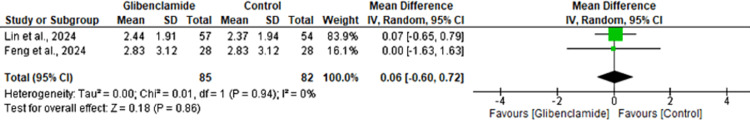
Meta-analysis of the mRS at three months between control patients and patients on glibenclamide [1,6] mRS: modified Rankin Scale

mRS at three months: Two studies assessed mRS at three months. Our meta-analysis demonstrated no significant difference between the glibenclamide and control group in mRS at three months (mean difference (MD): 0.06, 95% CI: -0.60 to 0.72, P = 0.86). The heterogeneity among studies was zero for mRS at three months (I² = 0%) (Figure 9).

Discussion

This meta-analysis synthesized evidence from randomized controlled trials to evaluate the efficacy and safety of glibenclamide in patients with aneurysmal subarachnoid hemorrhage (aSAH). While glibenclamide has shown mechanistic potential as a neuroprotective agent by inhibiting the SUR1-TRPM4 channel, a key mediator of cerebral edema, our findings demonstrate no statistically significant benefit of glibenclamide over controls in reducing delayed cerebral ischemia (DCI), hydrocephalus, and mortality or improving functional outcomes as measured by the modified Rankin Scale (mRS) at various time points. These results echo the challenges in translating preclinical promise into clinical efficacy, underscoring the need for further exploration of optimal dosing, timing, and formulation.

Our findings align with several studies on glibenclamide in other neurocritical conditions. For example, the Glyburide Advantage in Malignant Edema and Stroke (GAMES-RP) trial [8], which investigated intravenous glibenclamide in patients with malignant cerebral edema following large hemispheric infarction, failed to demonstrate significant improvements in primary outcomes, including mortality and functional status, although it did report reductions in midline shift. This suggests that while glibenclamide may exert some localized effects on edema, its impact on broader clinical outcomes remains unclear. The trial's focus on intravenous administration highlights a potential advantage in delivering consistent therapeutic levels, a factor that oral formulations may lack.

Similarly, the Glibenclamide in Aneurysmatic Subarachnoid Hemorrhage (GASH) study by da Costa et al. [4] assessed oral glibenclamide in aSAH patients and found no significant differences in mRS scores, mortality, or incidence of DCI compared to controls, despite employing an extended therapeutic window of 96 hours. Notably, our analysis mirrors these results, indicating limited clinical impact of oral glibenclamide even when treatment initiation is relatively early. This aligns with hypotheses that the pharmacokinetics of oral formulations, including variable absorption and potential sub-therapeutic concentrations at the site of injury, may hinder efficacy.

Preclinical studies have consistently demonstrated glibenclamide's ability to stabilize the blood-brain barrier (BBB), reduce cerebral edema, and attenuate inflammatory cascades. For instance, studies by Gerzanich et al. [9] and Sheth et al. [10] highlighted significant reductions in edema when glibenclamide was administered shortly after a neurological insult. These findings suggest that timing is critical, as the SUR1-TRPM4 channel, a primary target of glibenclamide, is most active during the early phases of cytotoxic and vasogenic edema [11]. Delayed administration, as seen in some clinical trials, may explain the diminished benefits observed in patient outcomes [1,5]. Furthermore, dosing strategies may play a pivotal role in determining efficacy. The GAMES-RP trial [8] utilized intravenous glibenclamide at doses designed to achieve optimal therapeutic levels without inducing hypoglycemia, while many studies investigating oral glibenclamide opted for lower doses to minimize adverse effects. This conservative dosing approach may compromise efficacy, particularly in severe cases of aSAH where higher drug concentrations at the site of injury are likely required.

Another relevant consideration is the variability in patient populations and concurrent treatments across studies. For instance, the GASH study [4] included patients treated with both endovascular and microsurgical approaches, which may influence outcomes such as DCI and functional recovery. Microsurgical interventions are often associated with higher risks of complications, potentially confounding the effects of glibenclamide [12]. Similarly, differences in baseline characteristics, such as the severity of aSAH or the presence of comorbidities, further complicate direct comparisons.

Although our findings and prior studies consistently indicate limited efficacy of glibenclamide in improving major clinical outcomes, certain trends warrant further investigation. For example, reductions in midline shift and edema volume observed in preclinical and smaller clinical studies suggest that glibenclamide may still hold promise as part of a multimodal treatment strategy [10]. Combining glibenclamide with other neuroprotective agents targeting complementary pathways, such as calcium channel blockers or anti-inflammatory therapies, could potentially enhance its therapeutic effects.

Hypoglycemia is a well-known adverse effect associated with the administration of sulfonylureas. The study by da Costa et al. [7] reported a hypoglycemia rate of 5.3%, which was comparable to that reported by Lin et al. [6] and Feng et al. [1] (8.7% and 14.2%, respectively). Our analysis showed no significant difference between the groups.

Current clinical guidelines for the management of aSAH, including those from the American Heart Association/American Stroke Association (AHA/ASA) and the Neurocritical Care Society, emphasize early aneurysm repair, maintenance of cerebral perfusion, and prevention of delayed cerebral ischemia (DCI) as primary goals of care [13,14]. The use of nimodipine remains the only pharmacological intervention with a class I recommendation for reducing poor outcomes associated with DCI [14]. Despite encouraging preclinical data, glibenclamide has not yet been incorporated into clinical guidelines for aSAH management due to a lack of robust evidence supporting its efficacy in improving mortality or functional outcomes. The 2023 AHA/ASA guidelines do not mention glibenclamide in their pharmacological recommendations for aSAH, underscoring the investigational status of this agent in clinical settings [15]. Similarly, European Stroke Organization (ESO) guidelines continue to focus on hemodynamic management, nimodipine, and timely surgical or endovascular intervention, with no current endorsement of sulfonylurea-based therapies [16]. As our meta-analysis and prior RCTs suggest, glibenclamide's clinical impact remains uncertain, and further large-scale trials are needed to clarify its role in guideline-directed management of aSAH.

The limited efficacy of oral glibenclamide in patients with aSAH may be partly attributed to its pharmacokinetic profile and restricted penetration across the blood-brain barrier [17]. In neurologically injured patients, oral drug absorption is often unpredictable due to impaired gastrointestinal motility and perfusion [18]. Furthermore, glibenclamide's BBB permeability is relatively low under normal conditions, and although BBB disruption after aSAH may enhance central drug delivery, the timing and extent of this disruption are variable [7,17]. Intravenous formulations, as used in ischemic stroke trials, achieve more consistent therapeutic levels and may offer better neuroprotective potential in acute central nervous system (CNS) injury settings.

Several limitations must be acknowledged. First, the small sample sizes across the included studies likely limited the statistical power to detect subtle but clinically meaningful differences. Second, heterogeneity in treatment protocols poses a challenge, such as the variability in dosing regimens (e.g., 5 mg versus 10 mg daily) and formulations (e.g., oral versus intravenous). Third, the potential for publication bias, as suggested by asymmetrical funnel plots for certain outcomes, warrants cautious interpretation.

## Conclusions

This systematic review and meta-analysis of randomized controlled trials demonstrated that glibenclamide does not confer statistically significant benefits in reducing the incidence of delayed cerebral ischemia, hydrocephalus, mortality, or hypoglycemia, nor does it improve functional outcomes measured by the modified Rankin Scale at discharge or follow-up intervals of three and six months in patients with aSAH. Future research should prioritize rigorously designed, multicenter RCTs that explore alternative delivery routes and standardized dosing protocols tailored to achieve effective CNS penetration without increasing adverse events such as hypoglycemia. Additionally, trials focusing on specific subgroups of aSAH patients may help identify populations most likely to benefit from glibenclamide. Evaluating the drug in combination with other neuroprotective interventions or surgical techniques may also yield synergistic effects worthy of exploration.

## Appendices

Table 3 presents the detailed search strategies employed in this study. 

**Table 3:** Search strategy used to identify related studies using different databases

Keywords	Databases
((Glibenclamide OR Glyburide OR Sulfonylurea) AND ("Subarachnoid Hemorrhage" OR "Aneurysmal Subarachnoid Hemorrhage" OR SAH OR "Cerebral Aneurysm Rupture"))	PubMed (26)
	Scopus (62)
	Central (10)
	Google Scholar (100)
	Web of Science (53)
	Total: 251

Table 4 presents the GRADE assessment for the evaluation of the reliability and certainty of the evidence presented in the included studies.

**Table 4:** GRADE assessment ^a^Substantial heterogeneity ^b^CI crossed the line of null effect GRADE: Grading of Recommendations, Assessment, Development, and Evaluations, CI: confidence interval, MD: mean difference

Certainty assessment							Number of patients		Effect		Certainty
Number of studies	Study design	Risk of bias	Inconsistency	Indirectness	Imprecision	Other considerations	Glibenclamide	Control	Relative risk (95% CI)	Absolute (95% CI)	
Risk of delayed cerebral ischemia											
2	Randomized trials	Not serious	Not serious	Not serious	Serious^b^	None	22/95	36/94	0.58 (0.31 to 1.10)	161 fewer per 1,000 (from 264 fewer to 38 more)	⨁⨁⨁◯ Moderate
Risk of hydrocephalus											
2	Randomized trials	Not serious	Not serious	Not serious	Serious^b^	None	27/85	16/82	1.65 (0.97 to 2.81)	127 more per 1,000 (from 6 fewer to 353 more)	⨁⨁⨁◯ Moderate
Risk of death											
2	Randomized trials	Not serious	Not serious	Not serious	Serious^b^	None	7/85	7/82	0.96 (0.35 to 2.66)	3 fewer per 1,000 (from 55 fewer to 141 more)	⨁⨁⨁◯ Moderate
Risk of hypoglycemia											
3	Randomized trials	Not serious	Not serious	Not serious	Serious^b^	None	11/123	4/122	2.19 (0.71 to 6.76)	39 more per 1,000 (from 10 fewer to 190 more)	⨁⨁⨁◯ Moderate
mRS at discharge											
2	Randomized trials	Not serious	Serious^a^	Not serious	Serious^b^	None	66	68	-	MD: -0.18 MD higher (-2.00 lower to 1.64 higher)	⨁⨁◯◯ Low
